# Physical Properties of Intact Proteins May Predict Allergenicity or Lack Thereof

**DOI:** 10.1371/journal.pone.0006273

**Published:** 2009-07-17

**Authors:** Suchita Singh, Bhupesh Taneja, Sundeep Santosh Salvi, Anurag Agrawal

**Affiliations:** 1 Institute of Genomics & Integrative Biology, Delhi, India; 2 Chest Research Foundation, Pune, India; LMU University of Munich, Germany

## Abstract

**Background:**

Predicting the allergenicity of proteins is challenging. We considered the possibility that the properties of the intact protein that may alter the likelihood of being taken up by antigen presenting cells, may be useful adjuncts in predicting allergens and non-allergens *in silico*. It has been shown that negatively charged acidic proteins are preferentially processed by dendritic cells.

**Methodology:**

Datasets (aeroallergen, food-allergen and non-allergen) for *in-silico* study were obtained from public databases. Isoelectric point (pI), net charge, and electrostatic potential (EP) were calculated from the protein sequence (for pI and net charge) or predicted structure (for EP).

**Result:**

Allergens and non allergens differed significantly in pI, net charge, and EP (p<0.0001). Cluster analysis based on these parameters resulted in well defined clusters. Non-allergens were characterized by neutral to basic pI (mean±SE, 7.6±0.16) and positive charge. In contrast allergens were acidic (5.7±0.15) and negatively charged. Surface electrostatic potentials calculated from predicted structures were mostly negative for allergens and mostly positive for non-allergens. The classification accuracy for non-allergens was superior to that for allergens. Thus neutral to basic pI, positive charge, and positive electrostatic potentials characterize non-allergens, and seem rare in allergens (p<0.0001). It may be possible to predict reduced likelihood of allergenicity in such proteins, but this needs to be prospectively validated.

## Introduction

Allergy and other forms of hypersensitivity affect up to 15–20% of the population in industrial nations [1], [2]. Several forms of this disorder are described and a major one is designated IgE-mediated allergy [3]. In general, the differentiating feature between an allergens and non-allergens is the formers ability to induce a specific IgE response via a series of complex interactions with the immune system including uptake, processing and recognition. Since the specific IgE immunoglobulins are directed at precise epitopes within the protein, they have received much attention. However, *in silico* evaluation of allergenicity of proteins, in terms of peptide motifs, has been relatively unsuccessful, and in spite of many efforts to define structural motifs that distinguish allergens from non allergenic proteins, there has been little progress in formulating structure activity relationships of allergenicity [4]. A possibility is that the B-cell and even T-cell epitopes that have been the focus of such efforts are only second-hand actors, depending upon uptake and processing of the protein prior to recognition [5]. The protein is first seen by antigen presenting cells (APCs), which must internalize whole molecules before cutting them into pieces. Physico-chemical properties of the protein such as lipid binding, ionic charge and post-translational modifications may be important in this process [6]. Lack of these features would be anticipated to reduce allergenicity even in the presence of structural epitopes that may otherwise predict allergenicity. It could then be modeled that potent allergens would possess both such properties as well as relevant epitopes; while non-allergens would lack either. While past efforts have focused on epitope mapping, bioinformatic investigations for discovery of physical properties of the whole protein that may underlie allergenicity are lacking in published literature.

Physical properties such as charge and pH have been shown to influence binding and uptake of ligands by antigen presenting cells (APC) [7]. Also, ions have important effects on respiratory health as well as particulate deposition and uptake patterns, and it is empirically known that electrical storms can precipitate asthma exacerbations [8]. From previous knowledge it has been found that electrostatic charge on inhaled particles can enhance and alter the level of deposition within the lung [9], [10]. Negatively charged particles may also experience stronger binding to the lung surface once deposited facilitating an increased allergic reaction [11]. Therefore we hypothesized that pH and/or ionic charge on the surface of proteins may be determinants or predictors of allergenicity, and tested this in an *in-silico* study.

## Results

### Allergens are acidic compared to non-allergens

Table 1 contains the summary data for 80 Aeroallergens, 50 Food-allergens and 80 Non-allergens whose complete data was available. (Full details are provided in supplement Data S1). It was found that aeroallergens were mostly acidic proteins (82% acidic; mean pI, 5.7), significantly different from non-allergens (20% acidic; mean pI, 7.6), but similar to food allergens (88% acidic; mean pI, 5.8). It followed that the calculated net charge for the entire protein in a neutral aqueous solution, was more negative for allergens compared to non-allergens.

**Table 1:** Calculated pI, net charge calculated from peptide sequence, net charge per amino acid, and a semiquantitative electrostatic potential score (EP) calculated from protein structure, are shown as mean±standard error mean (SEM) or median±interquartile range (IQR).

	Random-proteins		Aeroallergen		Food-allergen		Non-allergen		
	Mean (SEM)	Median (IQR)	Mean (SEM)	Median (IQR)	Mean (SEM)	Median (IQR)	Mean (SEM)	Median (IQR)	
pI	6.74 (0.19)	6.13 (2.86)	5.7*^§^ (0.16)	5.3 (1.4)	5.8*^§^ (0.16)	5.6 (1.2)	7.6^§^ (0.17)	7.7 (2.3)	P<0.0001 ^§^ <0.01
Net Charge	−3.76 (2.72)	−3 (14.25)	−6.32* (1.08)	−6.0 (8)	−5.9* (1.11)	−5 (5.7)	3.1 (2.31)	1 (6.25)	P<0.0001
Net Charge per aa	−0.01 (0.005)	−0.01 (0.05)	−0.04* (0.006)	−0.025 (0.052)	−0.02* (0.005)	−0.021 (0.05)	0.005 (0.004)	0.005 (0.022)	P<0.0001
EP Score	0.2 (0.25)	1.25 (3.5)	−0.5* (0.22)	−1.0 (2.9)	−0.5* (0.22)	−1.1 (2.7)	0.9 (0.28)	1.7 (4.1)	P<0.0001

Asterix (*) denotes significant differences when compared to non-allergens. (§) denotes significant difference from random proteins. There were no significant differences between aeroallergens or dietary allergens.

### Allergens have more negative electrostatic potential compared to non-allergens

The three dimensional electrostatic potential surface visualization of these proteins confirmed that there was an overall statistically significant tendency for allergens to have a negative electrostatic potential (83% of aeroallergens and 72% of food allergens versus 40% for non allergens, p<0.0001).

### Distinct clusters can be established for allergens and non-allergens

**Figure 1:**
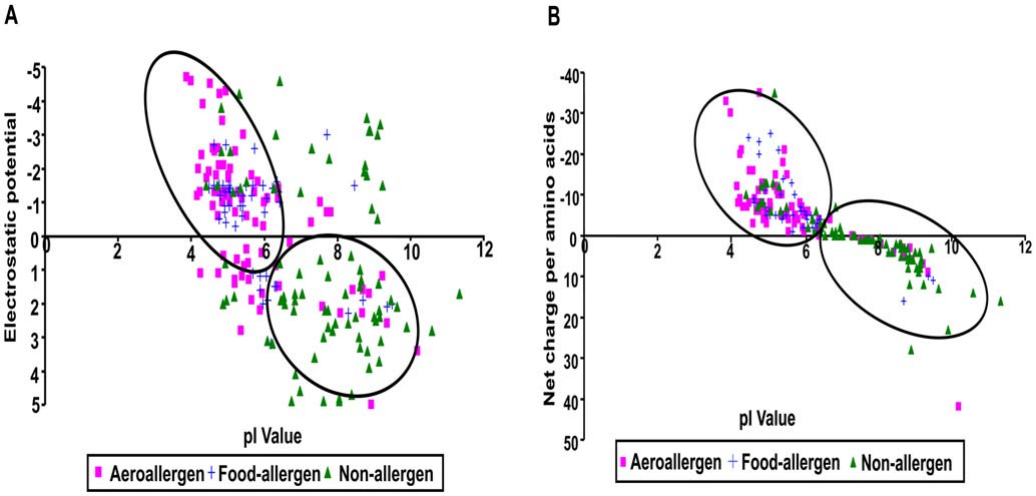
Two dimensional cluster analysis of allergens and non-allergens based on charge and pI. Square symbols denote aeroallergens, plus denotes food allergens, and inverted triangles denote non-allergens. pI is used as the X dimension in both analysis. The Y dimension is the (A) electrostatic potential score calculated from protein structure; and (B) net charge per amino acid calculated from peptide sequence.

Plots of the electrostatic potential (EP) scores and charge versus pI show distinct clusters for allergens and non-allergens with a classification accuracy of 93% (Figure 1). While the false classification rate was less than 10%, it was not possible to further distinguish aeroallergens from dietary allergens. Although, a three cluster approach (not shown) yielded an intermediate cluster enriched in dietary allergens but depleted in aeroallergens, there was a high rate of misclassification.

### The influence of charge and pI on allergenicity may be quantitative

**Figure 2:**
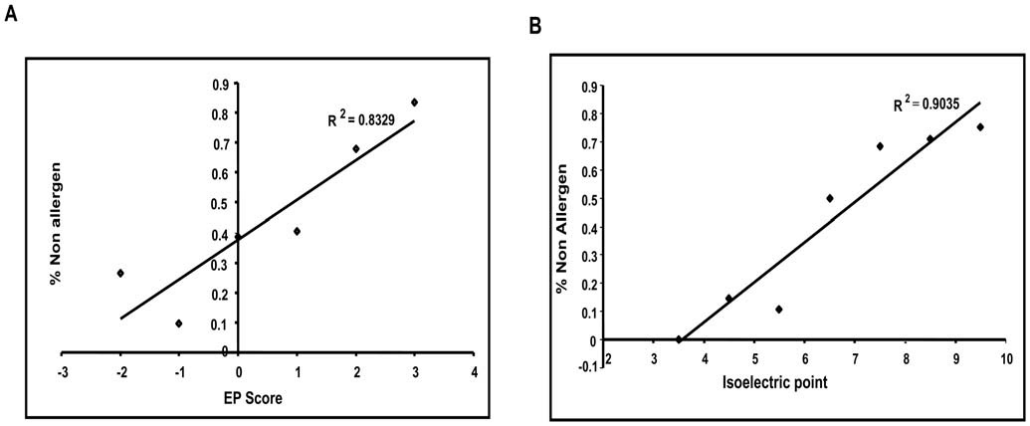
Linear trend analysis shows increasing proportions of non-allergens with increasing EP Score and pI. The proportion of non-allergens is shown as a linear function of surface electrostatic potential (A) and isoelectric point (B)

To assess whether a dose response relationship may exist between EP scores or pI and allergenicity, ordinal groups of proteins ranked by either EP scores or pI were analyzed for trends in proportions of allergens/non-allergens as described in materials and methods. Both pI and EP scores were positively correlated to the proportion of non-allergens (Figure 2). The linear by linear association trends of the chi square were extremely significant statistically with p values less than E-10.

## Discussion

In a simplified analysis of allergens for which three dimensional structure data could be either obtained or calculated, without accounting for post translational modifications, we found a highly significant tendency towards an acidic pI and a negative surface charge compared to non-allergens. We considered and rejected the possibility that overrepresentation of important allergen classes such as proteases may be confounding our data, because there was no significant bias towards acidic pI or negative surface charge for proteases as a class (data not shown). The proteins used for this study were taken from standard databases in a random fashion, and are expected to be representative of the groups from which they were drawn. This assumes that there was no relationship between the availability of protein sequence and structure and the parameters under study. In a random set of eighty proteins for which sequence and structure was available, there was neither any preponderance of acidic/basic proteins nor of negative/positive surface charge (Table 1), thereby supporting this assumption. Interestingly both allergens and non-allergens significantly differed from the control random set in pI, suggesting that neither lack of allergenicity nor high tendency towards it is the default norm. Thus, while the study was observational and speculative, the extreme differences in distribution suggest an underlying mechanistic process rather than a false association due to non-representative sampling.

This finding of negatively charged acidic proteins being overrepresented in the allergens could be understood in two contexts. First, it seemed possible that the processing of such proteins may be different. It has been previously shown that a population of T-cell may recognize immunogens based on their charge [12]. Negatively charged acidic proteins are preferentially taken up via scavenger receptors by macrophages and dendritic cells. While we present no direct experimental evidence of this, there is a published example in support. It has been shown recently that chemical modification of ovalbumin to increase the surface negative charge increased its antigenicity via efficient cross presentation by dendritics cells [13], [14]. Second, charge may have direct effects [15] on epithelium. Negative (but not positive) electrostatic charge on respired particles can activate the inflammatory vanilloid receptors and acid sensitive ions channels in human respiratory epithelial cells [16]. However, charge of soluble proteins is not directly comparable to charge on particulate matter and whether these receptors or channels can be activated by negative charged molecules is unknown. Interestingly, it has been postulated that one of the possible links between thunderstorms and asthma exacerbation could be electrostatically induced charge on allergens [8]. Although lightning strikes usually are negatively charged, the direction of electrostatic induction is unclear. Thus, the statistically increased frequency of negatively charged acidic proteins amongst allergens is most consistent with experimental evidence on uptake and processing of antigens by dendritic cells, although this too is speculative.

**Figure 3:**
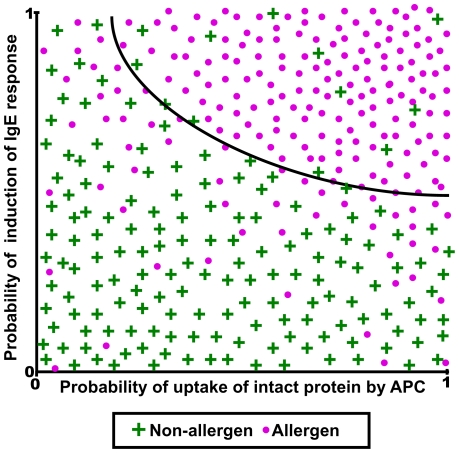
Schematic representation of the theoretical influence of physical properties of intact protein determining allergenicity. Probability of allergenicity is a cumulative function of probability of uptake (X axis) and probability of IgE induction by the processed protein (Y axis). It is conjectured that physical properties of the intact protein influence the probability of uptake.

How then do our results compare with the theoretical prediction of properties of the intact protein associated with allergen uptake (Figure 3), which would be necessary but not sufficient for allergenicity? Assuming that positive charge/neutral to basic pH as shown in Figure 2, corresponds to low probability of allergen uptake, the distributions noted by us are consistent with this. Few allergens (less than 10%) have positive charge/neutral to basic pH, while the converse is not true. It may therefore be possible to usefully predict low likelihood of allergenicity of unknown or modified proteins based on structural features. The quantitative dose effect of these variables as shown in Figure 2 supports this conclusion, especially for the extreme groups such as those with pI>8 and/or EP score>2. These numbers are only indicative based on our dataset and can be further improved as structural and sequence data for more proteins becomes available. A direct comparison with known *in-silico* tools for predicting allergens was not possible since we only used well known allergens in our allergens group and eliminated proteins with known similarity to allergens in our non-allergens group. Also, as discussed above, our findings only support supplemental benefit to existing tools. When we analyzed the list of clinically known allergens with Algpred, there were only seven misclassifications. Interestingly, six of these had acidic pI and five had negative potential, namely predictors of potential allergenicity by our analysis. Therefore, there may be some advantage to a combined analysis although given the very high rate of correct classification by Algpred for reasons discussed above, it was not statistically significant. Importantly, since specificity of the allergen predictions by existing methods is questionable, a published list of twenty-five clinically and experimentally defined non-allergens was used to explore the possibility that the use of physical properties of the whole protein may supplement the utility of epitope based prediction methods for non-allergens. Epitope or similarity analysis through Algpred (see materials and methods), revealed that 12 (48%) of these presumably non-allergenic proteins were predicted to be potential allergens. Ten of the 12 presumably wrongly predicted allergens had either positive surface potential or non-acidic pI, predictors of non-allergenicity in our analysis. A combined analysis would have misclassified only 2 of the 25 (8%) non-allergens from this list, a significant improvement over either method alone (48% for Algpred, 24% for physical properties). It would be interesting to repeat such analysis in more non-allergens that have not been pre-analyzed by *in-silico* epitope or similarity based methods. Also, other properties such as lipid binding that have been associated with allergenicity can be explored [17], [18]. However, given rare reports of possible allergy to most substances, defining non-allergens remains daunting.

Importantly, while this is bioinformatic data without direct experimental basis, the findings are in line with what is experimentally known about antigen uptake and processing in models. Unfortunately, it is not possible to take an unknown set of proteins and validate the predictions regarding any of them being non-allergenic. Non-allergens or rare-allergens can only be inferred by absence of any reports of allergenicity even after prolonged exposure to large number of people, such as during introduction of genetically modified food sources. Meanwhile, in conclusion, it can be said that physical properties of the intact protein are likely to be important determinants of allergenicity, and warrants consideration for incorporation into *in-silico* models.

## Materials and Methods

### Collection of allergens and non-allergens data

#### Dataset

The dataset used in this *in-silico* study were obtained from different public database based on the criterion of availability of structural data or possibility of prediction of structure from complete or near complete protein sequence based on homology.

### Aeroallergen Data

In this dataset of 80 proteins, 60 proteins were obtained from International union of immunological societies (I.U.I.S.) (www.allergen.org) [19]. Inclusion criteria were aeroallergen proteins with a sequence of greater than 50 amino acids and for which Protein Data Bank (PDB) structures were available. The other 20 proteins were obtained using the same criterion from SWISS-PROT Allergen Index (www.expasy.ch/cgi-bin/lists?allergen.txt).

### Food-allergen Data

A dataset of 50 food allergens were obtained from Swiss-Prot (www.expasy.ch/sprot/), International union of immunological societies (I.U.I.S.) (www.allergen.org), and Furmonaviciene and colleagues (http://www.nottingham.ac.uk/ immunology/research/BI)^4^. The selection criterions were otherwise identical to those for aeroallergens.

### Non-allergen Data

The (presumably) non allergenic protein dataset used in this study were obtained from Furmonaviciene and colleagues (http://www.nottingham.ac.uk/ immunology/research/BI) [4] and excerpted from commonly consumed commodities, such as rice, potato, cherry, apple, celery, tomato and salmon, to which allergies are rare or unknown. These species were selected to minimize the risk of introducing unknown allergens in this set. Selected proteins were carefully inspected to confirm lack of entries containing the strings ‘allergen’ or ‘allergy’ in any of the public databases mentioned previously. Other criterion such as a peptide sequence greater than 50 amino acids and presence of PDB structure were identical to allergens. It is noted that non-allergen does not necessarily imply “never-allergen” and could mean “rarely-allergen”, since absence of evidence is not evidence of absence.

### Data for Protein structure

Three dimensional protein structure models for aeroallergens, food-allergens and non allergens were obtained from PDB (Protein Data Bank) site www.rcsb.org.

### Data for the calculated parameters

For each protein, its PDB structure (Protein Databank), calculated pI (Isoelectric point), total amino acid content, total number of positively charged amino acid residues at neutral pH (7.0), total number of negatively charged amino acid residues at neutral pH, net charge at neutral pH (difference between positive and negative amino acids), and electrostatic potential were obtained. Calculated net charge being a function of the pI and the pH of the solution provided similar information to pI, albeit in a slightly different context. All the above data except electrostatic potential were obtained from ExPASy Proteomics server- ProtParam www.expasy.ch/tools/protparam.html, which allows the computational analysis of various physical and chemical parameters of proteins from its sequence.

### Calculation of electrostatic potential (EP)

Surface electrostatic potential was measured through online calculation by Protein Continuum electrostatics (PCE) web tool presently based on MEAD (macroscopic electrostatics with atomic detail) which generates electrostatic energies via finite difference solution to the Poisson–Boltzmann equation and can be accessed at http://bioserv.rpbs.jussieu.fr/cgi-bin/PCE-Pot, [20] and confirmed by offline calculation through Accelrys Discovery studio 2.0. These generate potentials and colored representations of electrostatic potentials mapped on the molecular surface. The proteins could be classified as positive or negative from this map, for analysis of proportions. For quantitative analysis, the colored representations of electrostatic potentials mapped on the molecular surface were numerically represented by the freely downloadable ImageJ software (National Institutes of Health, http://rsbweb.nih.gov/ij/). An empiric ordinal scale (EP score) ranging from −5 to +5 representing a series of admixtures of negative and positive potentials (−5, 100/0; −4, 90/10; −3, 80/20; −2, 70/30; −1, 60/40; 0, 50/50; +1, 40/60; +2, 30/70; +3, 20/80; +4, 10/90, +5, 0/100) was used since EPs are calculated at a point in space and summation over a region is not valid.

### Prediction of allergenicity *In silico*

We used the tool AlgPred: Prediction of Allergenic protein and mapping of IgE Epitopes developed by Saha et al (http://www.imtech.res.in/raghava/algpred/index.html) [21] for determining whether proteins sequences contain any known IgE epitope or any similarity with allergen sequences.

### Statistics

Mean±standard deviations and median±inter quartile were calculated for all parameters. Means were compared by unpaired t-tests and proportions were compared by chi square test. Trends in proportions were analyzed by calculating the Pearson's correlation coefficient (r^2^) of a plot of linearly arranged ordinal groups of the dependent variable, followed by calculation of the associated linear by linear association probability of the chi statistic (M^2^ = (n−1)×r^2^; df = 1, where M^2^ is the chi statistic, n is sample size, r is correlation coefficient) [22].

## Supporting Information

Data S1 Data file in MS Excel format
(0.06 MB XLS)
